# A kinetic study for the Fenton and photo-Fenton paracetamol degradation in an annular photoreactor

**DOI:** 10.1007/s11356-018-3098-4

**Published:** 2018-09-18

**Authors:** Francesca Audino, Leandro Oscar Conte, Agustina Violeta Schenone, Montserrat Pérez-Moya, Moisès Graells, Orlando Mario Alfano

**Affiliations:** 1 Chemical Engineering Department, Universitat Politècnica de Catalunya, Escola d’Enginyeria de Barcelona Est (EEBE), Av. Eduard Maristany, 16, 08019 Barcelona, Spain; 2 Instituto de Desarrollo Tecnológico para la Industria Química (INTEC), Consejo Nacional de Investigaciones Científicas y Técnicas (CONICET) and Universidad Nacional del Litoral (UNL), 3000 Santa Fe, Argentina

**Keywords:** AOPs, Pharmaceuticals, Annular photoreactor, Design of experiments, LVRPA, Kinetic modeling

## Abstract

A kinetic model describing Fenton and photo-Fenton degradation of paracetamol (PCT) and consumption of hydrogen peroxide (H_2_O_2_) was proposed. A set of Fenton and photo-Fenton experiments (18 runs in total) was performed by fixing the initial concentration of PCT to 40 mg L^−1^ and varying the initial concentrations of H_2_O_2_ and ferrous ion, Fe^2+^. The experimental set-up was a well-stirred annular photoreactor equipped with an actinic BL TL-DK 36 W/10 1SL lamp. Experimental results highlighted that PCT is no more detected by HPLC analysis within a minimum reaction time of 2.5 and a maximum reaction time of 15.0 min. Besides, a maximum conversion of total organic carbon (TOC) of 68.5% was observed after 75 min of reaction in case of using UV radiation and the highest concentrations of the Fenton reagents. The experimental data were used to fit the kinetic model. The radiation field inside the reactor was taken into account through the local volumetric rate of photon absorption, evaluated by assuming a line source model with spherical and isotropic emission. The kinetic parameters were estimated by using a non-linear least-squares regression procedure and root mean square errors (RMSE) were calculated in order to validate the feasibility of the proposed model. A good agreement between experimental and predicted data was observed and the lowest values of RMSE resulted in 5.84 and 9.59% for PCT and H_2_O_2_ normalized concentrations, respectively.

## Introduction

In the last decades, a notable effort has been made to investigate the main aspects of the advanced oxidation processes (AOPs). AOPs are a group of chemical processes all characterized by the capability of exploiting the high reactivity of HO^∙^radicals in driving oxidation reactions that are suitable for achieving pollutant remediation and mineralization (Andreozzi et al. 1999). Hence, AOPs represent a convenient application to wastewater treatment, also considering the possibility to combine the biological treatment with an oxidative degradation of toxic or refractory substances (Oller et al. 2011).

Especially, they can be adopted for the treatment of the so-called contaminants of emerging concern (CECs). CECs are a group of chemicals, including pharmaceuticals and personal care products (PPCPs), which are increasingly being detected at low concentrations (ng L^−1^ to μg L^−1^) in surface water, ground waters, and even drinking waters, and that may be included in future environmental regulations depending on the results of the investigations on their effects on the human health and environment (EPA, nd). This shows that conventional sewage treatment plants are not able to remove this kind of contaminants.

According to the current legislation, the Directive 2013/39/EU of the European Parliament and of the Council of 12 August 2013 amending Directives 2000/60/EC and 2008/105/EC, an important factor to be taken into account is the CEC monitoring as well as the reinforcement of the risk assessment of pharmaceutical products (Ribeiro et al. 2015).

Regarding pharmaceuticals, in the last two decades, a large variety of drugs (analgesics, anti-inflammatory, antibiotics, etc.) coming from domestic, industrial, hospital, and health center waste waters or from landfill leachates, have been detected in soils, surface waters, ground waters, and drinking waters.

Particularly, the photo-enhanced Fenton process has proved to be highly efficient in degrading CECs (Miralles-Cuevas et al. 2014) as well as strength organic wastewaters (Pouran et al. 2015). As a matter of fact, the review by Wang et al. (2016) highlighted several industrial applications of the photo-Fenton process that has been used to treat different kinds of wastewaters such as olive-oil mill, textile, pesticide, cosmetic, dye, fermentation brine, green olives, pharmaceutical, cork cooking, pulp mill, and phenolic wastewaters.

The Fenton reaction is an old reactive system proposed by Fenton in 1894 (Fenton 1894), that occurs by means of addition of hydrogen peroxide (H_2_O_2_) to ferrous ion salts (Fe^2+^) and that leads to the formation of hydroxyl radicals, mainly HO˙. The photo-assisted Fenton process (Kiwi et al. 1993; Pulgarin and Kiwi, 1996) represents an extension of the Fenton process obtained by using a UV-VIS light source. Under irradiated conditions and acid medium (pH = 2.8), the photolysis of ferric ion (Fe^3+^) complexes (Fe(OH)^2+^) occurs, allowing the Fe^2+^ regeneration and the formation of an additional hydroxyl radical leading to a strong increase of the degradation rate of organic pollutants.

A large experimental effort has led to an extensive knowledge on photo-Fenton process. Several works, at both laboratory and pilot plant scale, have investigated the key process efficiency parameters, such as H_2_O_2_ consumption, processing time, and mineralization rate, as well as the effect of factors like temperature, pH, dissolved ion concentration, and dissolved organic carbon (DOC) on such parameters (Andreozzi et al. 2000; Pignatello et al. 2006; Farias et al. 2007; Zapata et al. 2010). Conversely, despite the extensive experimental work, the mathematical modeling is still under development. Specifically, concerning photo-Fenton kinetics modeling, mainly three different approaches have been proposed: first principles models (FPMs), empirical models (EMs), and data based models (DBMs). FPMs rely on the description of all the elementary steps of the process. The works by Kang et al. (2002), Jeyong and Yoon (2005), and Ortiz de la Plata et al. (2010) are representative examples of FPMs. On the other hand, EMs rely on a complete empirical approach based on the use of regression models (Kusic et al. 2006) eventually coupled with the design of experimental techniques (Pérez-Moya et al. 2008). Finally, in the area of DBMs, artificial neural networks (ANN) (Göb et al. 2001), support vector regression (SVR) (Shokry et al. 2015), and ordinary kriging (OK) (Shokry et al. 2015) have been used. While currently EMs and DBMs are unable to fully capture the complexity and nonlinear nature of such processes, the accuracy and understanding that might provide FPMs are unaffordable. Moreover, few works have addressed the modeling of the radiation field inside the photoreactor that requires the evaluation of the local volumetric rate of photon absorption (LVRPA) (Cassano et al. 1995; Conte et al. 2016).

Thus, the present study aims at presenting a kinetic model that can be a compromise solution between the unaffordable complexity of the FPMs and the oversimplification of the EMs and DBMs, without disregarding the assessment of the LVRPA effect.

Paracetamol (PCT) was selected as model pollutant since it is one of the top 200 pharmaceuticals prescribed overall the world, being widely used as antipyretic and analgesic. Therefore, PCT is continuously released by hospital waste (Langford and Thomas, 2009), as well as by consumer use and disposal (according to Muir et al. 1997, PCT is excreted in 58–68% during therapeutic treatment). As a consequence, it has been detected in the effluents of sewage treatment plants in μg L^−1^ (Ternes, 1998), in natural water sources at concentrations higher than 65 μg L^−1^ in the Tyne River (UK) (Antunes et al. 2013), and even in groundwater at concentration ranging between μg L^−1^ and ng L^−1^ (De Gusseme et al., 2011).

However, another issue is the treatment of real paracetamol wastewaters that are characterized by high levels of PCT, total organic carbon (TOC), and chemical oxygen demand (COD) concentrations. A recent work by Dalgic et al. (2017) showed that the Fenton process can be an effective pre-treatment of a real paracetamol wastewater of the pharmaceutical industry characterized by a PCT concentration between 37 and 294 mg L^−1^. Previously, Roshanfekr Rad et al. (2015) also investigated the use of photo-Fenton process in industrial applications. Particularly, these authors analyzed the effect of different operational parameters on the photo-Fenton process including the phenol and paracetamol initial concentrations ranging between 20 and 100 mg L^−1^. Cabrera Reina et al. (2012) proposed a model to track the photo-Fenton degradation of paracetamol present at high initial concentration (4–25 mmol L^−1^ of TOC) or rather simulating an industrial wastewater.

In the present work, the experimental data set used to study the kinetic model was based on an initial concentration of PCT ($\left[{C}_{\mathrm{PCT}}^{t0}\right]$) of 40 mg L^−1^ (e.g., industrial wastewater). The initial H_2_O_2_ concentration ($\left[{C}_{{\mathrm{H}}_2{\mathrm{O}}_2}^{t0}\right]$) was varied between half and twice the stoichiometric dose to achieve mineralization of 40 mg L^−1^ of PCT and the initial ferrous ion concentrations ($\left[{C}_{{\mathrm{Fe}}^{2+}}^{t0}\right]$) between 5 and 10 mg L^−1^ (being the latter the maximum value allowed in wastewaters in Spain).

Reaction rates for PCT and H_2_O_2_ were obtained and then ordinary differential equations (ODEs) were used for describing component mass balances inside the reactor. A line source radiation model with spherical and isotropic emission (LSSE model) was adopted in order to derive the equation describing the variation of LVRPA with the absorbing species.

Experimental and predicted concentrations of PCT and H_2_O_2_ were compared by implementing a non-linear least-squares regression procedure in MATLAB and root mean square errors (RMSE) were calculated to test model reliability.

## Methodological framework: experimental settings and process modeling

The general methodology consists of an experimental part and a modeling section including model fitting (parameter estimation), that are detailed in the following sections.

### Experimental

The experimental step provides the data that will be used in the final optimization procedure to estimate the kinetic constants of the proposed model.

#### Reagent and chemicals

Paracetamol 98% purity purchased from Sigma-Aldrich was used as model pollutant. Reagent-grade hydrogen peroxide 33% *w*/*v* from Panreac and iron sulfate (FeSO_4_·7H_2_O) from Merck adopted as the ferrous ion (Fe^2+^) source, were used to perform all the experiments. HPLC gradient grade methanol, MeOH, purchased from J.T. Baker and filtered Milli Q grade water were used as HPLC mobile phases. High-purity (> 99%) ascorbic acid from Riedel de Haën, 0.2% 1,10-phenanthroline from Scharlab, and sodium acetate anhydrous and 95%–98% sulfuric acid, both from Panreac, were used to perform iron species measurements. In order to adjust the initial pH to the optimal one (2.8 ± 0.1), hydrogen chloride HCl 37% from J.T. Baker was used. Distilled water was used as water matrix in all experiments.

#### Analytical determinations

Measurements of PCT, total organic carbon (TOC), H_2_O_2_, and iron species concentrations were performed. TOC concentration (*C*_TOC_) was measured with a Shimadzu VCHS/CSN TOC analyzer and samples were taken each 15 min until the end of the experiment. PCT concentration (*C*_PCT_) was determined using an HPLC Agilent 1200 series with UV-DAD. The measurement method is the one described by Yamal-Turbay et al. (2014). All the samples, taken at 0, 1.5, 2.5, 5, 7.5, 10, and 15 min, were treated with 0.1 M methanol (in proportion 50:50) to stop reaction and further degradation of PCT. Hydrogen peroxide concentration (${C}_{{\mathrm{H}}_2{\mathrm{O}}_2}$) was determined with a Hitachi U-2001 UV-VIS spectrophotometer and using the spectrophotometric technique described by Nogueira et al. (2005). This technique is based on the measurement of the absorption at 450 nm of the complex formed after reaction of H_2_O_2_ with ammonium metavanadate. In this case, samples were taken each 5 min until a reaction time of 30 min and then each 15 min until the end of the assay.

The iron species (Fe^2+^, Fe^TOT^) were analyzed using the 1,10-phentranoline method following ISO 6332 (ISO 6332:^1988^), based on the absorbance measurements of the Fe^2+^-phenantroline complex at 510 nm. To measure total iron concentration (${C}_{{\mathrm{Fe}}^{\mathrm{TOT}}}$), ascorbic acid must be used so to convert all the ferric ions (Fe^3+^) to ferrous ions (Fe^2+^). Then, for difference, ferric ion concentration could be determined (${C}_{{\mathrm{Fe}}^{3+}}$ = ${C}_{{\mathrm{Fe}}^{\mathrm{TOT}}}$-${C}_{{\mathrm{Fe}}^{2+}}$). In this case, samples were taken each 5 min until a reaction time of 30 min and then each 15 min until the end of the assay.

Table 1 shows the experimental errors evaluated for all measurement techniques and for a PCT concentration range of [0–40] mg L^−1^, a total carbon (TC) concentration range of [0–50] mg L^−1^, an inorganic carbon (IC) concentration range of [0–10] mg L^−1^, and a H_2_O_2_ concentration range of [0–150] mg L^−1^.

**Table 1:** Experimental errors of the measurement techniques

Measurement equipment	Measured species	Error (mg L^−1^)
HPLC Agilent 1200 series with UV-DAD	PCT [0–40] mg L^−1^	0.15
Shimadzu VCHS/CSN TOC analyzer	TC [0–50] mg L^−1^	0.23
	IC [0–10] mg L^−1^	0.04
Hitachi U-2001 UV-Vis spectrophotometer	H_2_O_2_ [0–150] mg L^−1^	1.43

#### Experimental set-up

**Fig. 1:**
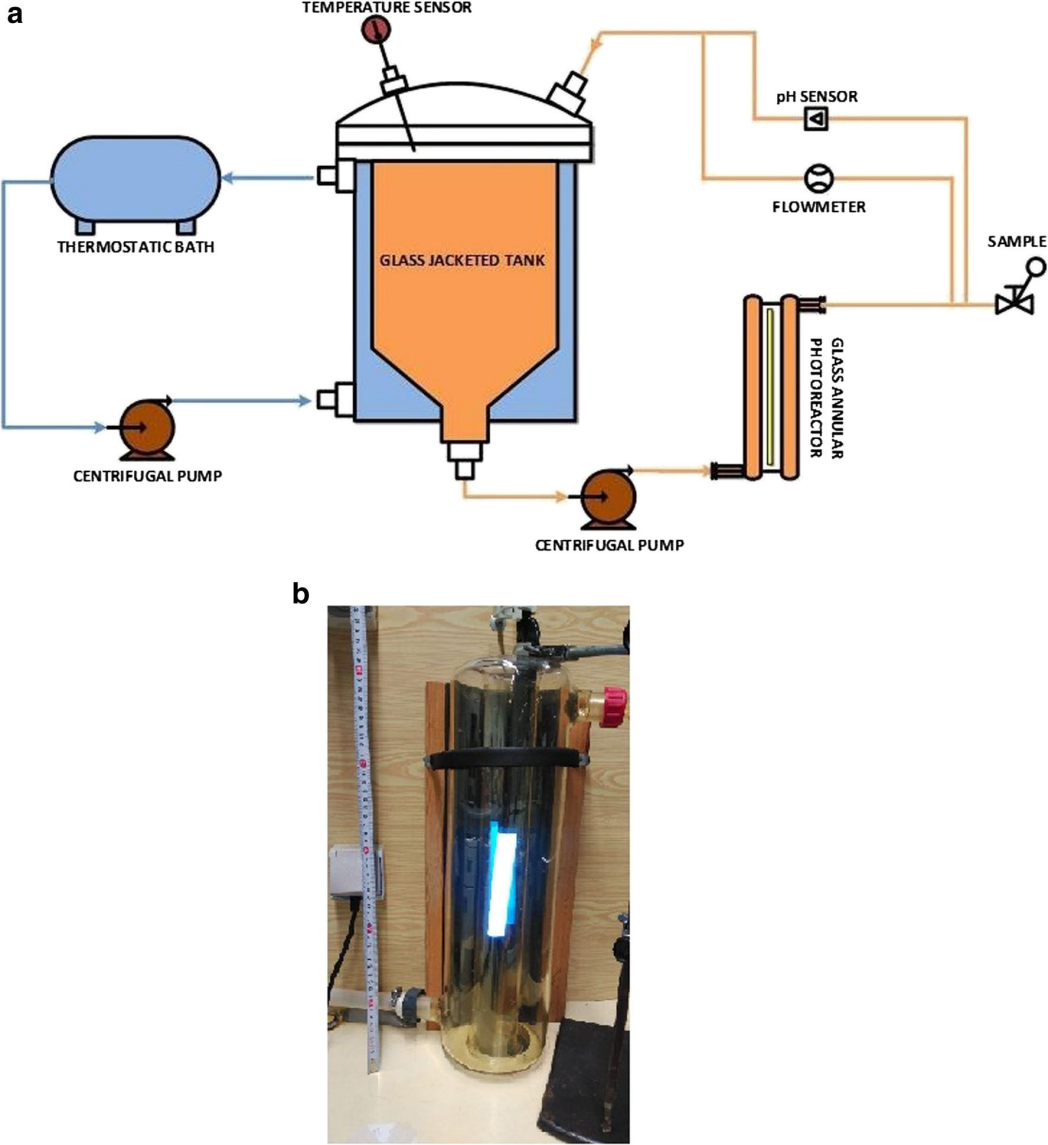
Experimental set-up. **a** Schematic view. **b** Picture

A 15-L system composed by a 9-L glass jacketed reservoir tank and a 6-L glass annular photoreactor equipped with an Actinic BL TL-DK 36 W/10 1SL lamp (UVA-UVB) was used to perform Fenton and photo-Fenton experiments; the irradiated volume is 10% of the total volume (that is 1.5 L). The incident photon power, *E* = 3.36 × 10^−4^ Einstein min^−1^ (300 and 420 nm) was measured by Yamal-Turbay et al., 2014 using potassium ferrioxalate actinometry (Murov et al. 1993). In addition, the experimental device is also equipped with a pH sensor and a flowmeter for the control of the recirculation flow rate and a thermostatic bath for the temperature control, which is measured by a temperature sensor placed inside the 9-L tank. In Fig. 1, a schematic view (Fig. 1a) and a picture of the experimental set-up (Fig. 1b) with its specifications (Table 2) are shown. For more details of the experimental system, you can refer to Yamal-Turbay et al. (2014).

**Table 2:** Experimental device specifications

Tank reactor		
Total volume, L		9
Annular reactor		
Total volume, L		6
Irradiated volume, L		1.5
Annular irradiated height, mm		130
Outer cylinder	Outer diameter, mm	150
	Inner diameter, mm	140
Inner cylinder	Outer diameter, mm	70
	Inner diameter, mm	63.6
Irradiation system		
Actinic BL TL-DK 36 W/10 1SL		
Diameter, mm		28
Length, mm		589.8

#### Experimental procedure

Fenton and photo-Fenton assays were performed in batch mode with recirculation, changing initial concentrations of hydrogen peroxide (${C}_{{\mathrm{H}}_2{\mathrm{O}}_2}^{t0}$) and ferrous ion (${C}_{{\mathrm{Fe}}^{2+}}^{t0}$) for the same value of the initial concentration of PCT (${C}_{\mathrm{PCT}}^{t0}$).

The value of the initial PCT concentration was set to 40 mg L^−1^ in order to investigate Fenton and photo-Fenton treatment of a real paracetamol wastewater characterized by higher PCT concentrations (Dalgic et al. (2017)).

The maximum value of the initial concentration of Fe^2+^ was set taking into account the maximum legal value in wastewaters in Spain (DOGC, 2003), 10 mg L^−1^, while half of such value was set as the minimum value to be investigated.

Also, to select the initial concentration of H_2_O_2_, the stoichiometric dose to achieve total mineralization when H_2_O_2_ is considered to be the only oxidant in the media (Eq. (1)) and when ${C}_{\mathrm{PCT}}^{t0}$ = 40 mg L^−1^, was calculated and resulted in a value of 189 mg L^−1^. Then, a range between half and twice the stoichiometric dose (94.5 and 378 mg L−1, respectively) was selected.

1 $$ {\mathrm{C}}_8{\mathrm{H}}_9{\mathrm{NO}}_2+21{\mathrm{H}}_2{\mathrm{O}}_2\to 8{\mathrm{C}\mathrm{O}}_2+25{\mathrm{H}}_2\mathrm{O}+{\mathrm{H}}^{+}+{\mathrm{NO}}_3^{-} $$

Eighteen experiments were carried out (Table 3) by testing three different values of ${C}_{{\mathrm{H}}_2{\mathrm{O}}_2}^{t0}$ (94.5, 189, and 378 mg L^−1^) and ${C}_{{\mathrm{Fe}}^{2+}}^{t0}$(5, 7.5, and 10 mg L^−1^), for the same value of ${C}_{\mathrm{PCT}}^{t0}$ set to 40 mg L^−1^ (corresponding to ${C}_{\mathrm{TOC}}^{t0}$ = 25.40 mg L^−1^) and under dark and irradiated conditions. Hence, three hydrogen peroxide to paracetamol initial molar ratios (*R* = 10.5, 21, 42) were investigated.

**Table 3:** Design of experiments

*ID*	${C}_{{\mathrm{Fe}}^{2+}}^{t0}$ (mg L^−1^)	${C}_{{\mathrm{H}}_2{\mathrm{O}}_2}^{t0}$ (mg L^−1^)	Irradiation	R (−) H_2_O_2_:PCT
E1	5	94.5	No	10.5
E2	5	189	No	21
E3	5	378	No	42
E4	7.5	94.5	No	10.5
E5	7.5	189	No	21
E6	7.5	378	No	42
E7	10	94.5	No	10.5
E8	10	189	No	21
E9	10	378	No	42
E10	5	94.5	Yes	10.5
E11	5	189	Yes	21
E12	5	378	Yes	42
E13	7.5	94.5	Yes	10.5
E14	7.5	189	Yes	21
E15	7.5	378	Yes	42
E16	10	94.5	Yes	10.5
E17	10	189	Yes	21
E18	10	378	Yes	42

Regarding the experimental protocol, the glass reservoir was first filled with 10 L of distilled water and then, after 15 min of recirculation, 4.9 L of distilled water in which PCT was previously dissolved, were added. Once pH was adjusted to 2.8 ± 0.1, the remaining 0.1 L of distilled water, in which Fe^2+^ was previously dissolved, were filled and the light was switched on (in case of experiments performed under irradiated conditions). Finally, H_2_O_2_ was added and the initial sample was taken. The total reaction time was fixed to 120 min. To ensure perfect mixing conditions, according to the results obtained by Yamal-Turbay et al. (2014), the recirculation flow rate was set to 12 L min^−1^.

### Modeling

The modeling section starts by proposing a kinetic model followed by the reactor model that allows describing, by the means of a set of ODEs, the mass balances into the photoreactor (isothermal conditions).

#### Kinetic model

The kinetic model proposed for the Fenton and photo-Fenton degradation of PCT (see Table 4) is based on the general Fenton/photo-Fenton reaction scheme proposed by Sun and Pignatello (1993a,b); Brillas et al.(2000); and Pignatello et al.(2006).

**Table 4:** Reaction mechanism of Fenton and photo-Fenton PCT degradation

ID	Reaction steps	Kinetic constants
1	${\mathrm{Fe}}^{2+}+{\mathrm{H}}_2{\mathrm{O}}_2\overset{k_1}{\to }{\mathrm{Fe}}^{3+}+{\mathrm{H}\mathrm{O}}^{-}+{\mathrm{H}\mathrm{O}}^{\bullet }$	*k*_1_
2	${\mathrm{Fe}}^{3+}+{\mathrm{H}}_2\mathrm{O}\overset{\overline{\varPhi}}{\to }{\mathrm{Fe}}^{2+}+{\mathrm{H}}^{+}+{\mathrm{H}\mathrm{O}}^{\bullet }$	$\overline{\varPhi}$
3	${\mathrm{Fe}}^{3+}+{\mathrm{H}}_2{\mathrm{O}}_2\overset{k_3}{\to }{\mathrm{Fe}}^{2+}+{\mathrm{H}}^{+}+{\mathrm{H}\mathrm{O}}_2^{\bullet }$	*k*_3_
4	${\mathrm{H}}_2{\mathrm{O}}_2+{\mathrm{H}\mathrm{O}}^{\bullet}\overset{k_4}{\to }{\mathrm{H}\mathrm{O}}_2^{\bullet }$+ H_2_O	*k*_4_
5	$\mathrm{PCT}+{\mathrm{HO}}^{\bullet}\overset{k_5}{\to }{P}_i$	*k*_5_

Where $\overline{\ \varPhi }$ refers to the wavelength-averaged primary quantum yield that was taken from Bossmann et al. (1998) and *P*_*i*_ represents the generic intermediate compound generated by the hydroxyl radical (*HO*^∙^) attack to PCT.

The proposed model is based on the following assumptions (Conte et al. 2012):

i. only the hydroxyl radicals (HO^∙^) are taken into account as oxidant species;
ii. the steady-state approximation (SSA) can be applied to the highly reactive species (HO^∙^);
iii. low ferrous ion concentrations were selected so the hydroxyl radical attack to Fe^2+^ can be neglected;
iv. the radical-radical termination steps are negligible compared to the propagation steps;
v. the oxygen concentration is always in excess.

Hence, the kinetic constants accounting for Fenton and Fenton-like reactions,and the hydroxyl radical attack to hydrogen peroxide and paracetamol (*k*_1_, *k*_3_, *k*_4_, and *k*_5_, respectively) were the parameters to be estimated. Subsequently, the following reaction rates for the reactive species PCT, H_2_O_2,_ Fe^2+^, and Fe^3+^ were derived:

2 $$ \left[\begin{array}{c}{R}_{\mathrm{PCT}}\left(\underset{\_}{x},t\right)\\ {}{R}_{{\mathrm{H}}_2{\mathrm{O}}_2}\left(\underset{\_}{x},t\right)\\ {}{R}_{{\mathrm{Fe}}^{2+}}\left(\underset{\_}{x},t\right)\\ {}{R}_{{\mathrm{Fe}}^{3+}}\left(\underset{\_}{x},t\right)\end{array}\right]=\left[\begin{array}{c}{R^T}_{\mathrm{PCT}}\left(\underset{\_}{x},t\right)\\ {}{R^T}_{{\mathrm{H}}_2{\mathrm{O}}_2}\left(\underset{\_}{x},t\right)\\ {}{R^T}_{{\mathrm{Fe}}^{2+}}\left(\underset{\_}{x},t\right)\\ {}{R^T}_{{\mathrm{Fe}}^{3+}}\left(\underset{\_}{x},t\right)\end{array}\right]+\overline{\varPhi}\sum \limits_{\lambda }{e}_{\lambda}^a\left(\underset{\_}{x},t\right)\left[\begin{array}{c}-\frac{1}{\delta}\\ {}-\frac{1}{\rho}\\ {}\kern1em 1\\ {}-1\end{array}\right] $$

Where

3 $$ {\displaystyle \begin{array}{c}\ \\ {}\delta =\frac{k_4}{k_{12}}\frac{\ {C}_{{\mathrm{H}}_2{\mathrm{O}}_2}}{\ {C}_{\mathrm{PCT}}}\kern0.75em +1\end{array}} $$

4 $$ \rho =\frac{k_5}{k_4}\frac{\ {C}_{\mathrm{PCT}}}{\ {C}_{{\mathrm{H}}_2{\mathrm{O}}_2}}\kern0.75em +1 $$

Here, $\overline{\ \varPhi }$ is the wavelength-averaged primary quantum yield,$\sum \limits_{\lambda }{e}_{\lambda}^a\left(\underset{\_}{x},t\right)$ the *LVRPA* extended to polychromatic radiation by performing the integration over all useful wavelengths *λ* (300–420 nm), and $\underset{\_}{x}$ the position vector accounting for the radius and axial coordinates of the reactor.

It should be noted that the general reaction rate expression can be expressed as follows (in matrix notation):

5 $$ \left[\boldsymbol{R}\left(\underset{\_}{x},t\right)\right]=\left[{\boldsymbol{R}}^T\left(\underset{\_}{x},t\right)\right]+\overline{\varPhi}\sum \limits_{\lambda }{e}_{\lambda}^a\left(\underset{\_}{x},t\right)\boldsymbol{\tau} \left(\underset{\_}{x},t\right) $$

The first term on the right-hand side of Eq. (2) corresponds to the thermal reaction rate that gives the *i*-component degradation by the Fenton reaction, taking place in the total volume *V*_*T*_, and is given by Eqs. (6) and (7):

6 $$ \left[\begin{array}{c}{R}_{\mathrm{PCT}}^T\left(\underset{\_}{x},t\right)\\ {}{R}_{{\mathrm{H}}_2{\mathrm{O}}_2}^T\left(\underset{\_}{x},t\right)\\ {}{R}_{{\mathrm{Fe}}^{2+}}^T\left(\underset{\_}{x},t\right)\\ {}{R}_{{\mathrm{Fe}}^{3+}}^T\left(\underset{\_}{x},t\right)\end{array}\right]={k}_1\ {C}_{{\mathrm{Fe}}^{2+}}\ {C}_{{\mathrm{H}}_2{\mathrm{O}}_2}\left[\begin{array}{c}\kern1.25em -\frac{1}{\delta}\\ {}-\left(1+\frac{1}{\rho}\right)\\ {}\kern0.75em -1\\ {}\kern0.75em +1\end{array}\right]+\gamma \left[\begin{array}{c}\kern2em 0\kern1.25em \\ {}-1\\ {}+1\\ {}-1\end{array}\right] $$

where:

7 $$ \gamma ={k}_2\ {C}_{{\mathrm{Fe}}^{3+}}\ {C}_{{\mathrm{H}}_2{\mathrm{O}}_2} $$

On the other hand, the second term on the right-hand side of Eq. (2) corresponds to the *i*-component degradation by the radiation-activated reaction occurring inside the irradiated liquid volume (*V*_IRR_).

#### Reactor model

The mass balances and initial conditions for the well-stirred annular photoreactor are given by the following set of first order, ordinary differential equations:

8 $$ \frac{d\boldsymbol{C}}{dt}={\boldsymbol{R}}^T(t)+\frac{V_{\mathrm{IRR}}}{V_T}\overline{\varPhi}{\left\langle \sum \limits_{\lambda }{e}_{\lambda}^a\left(\underset{\_}{x},t\right)\right\rangle}_{V_{\mathrm{IRR}}}\boldsymbol{\tau} \left(\underset{\_}{x},t\right) $$

With the initial conditions:

9 $$ \boldsymbol{C}={\boldsymbol{C}}^0\kern0.5em {t}_0=0 $$

Note that the required reaction rate expressions to be replaced in Eq. (8) are given by Eqs. (2)–(4) and Eqs. (6)–(7).

Therefore, based on the previous considerations, the following ODEs system gives the mass balance equations of the reactor model for each species (PCT, H_2_O_2_, Fe^2+^, and Fe^3+^):

10 $$ \frac{d{C}_{PCT}}{dt}=\left[\left({k}_1\ {C}_{{\mathrm{Fe}}^{2+}}\ {C}_{{\mathrm{H}}_2{\mathrm{O}}_2}\left(-\frac{1}{\delta}\right)\right)\right]+\left[\frac{V_{\mathrm{IRR}}}{V_T}\left(\overline{\varPhi}{\left\langle \sum \limits_{\lambda }{e}_{\lambda}^a\left(\underset{\_}{x},t\right)\right\rangle}_{V_{\mathrm{IRR}}}\right)\right] $$

11 $$ \frac{d{C}_{{\mathrm{H}}_2{\mathrm{O}}_2}}{dt}=\left[\left({k}_1\ {C}_{{\mathrm{Fe}}^{2+}}\ {C}_{{\mathrm{H}}_2{\mathrm{O}}_2}\left(-\left(1+\frac{1}{\rho}\right)\right)-\gamma \right)\right]+\left[\frac{V_{\mathrm{IRR}}}{V_T}\left(\overline{\varPhi}{\left\langle \sum \limits_{\lambda }{e}_{\lambda}^a\left(\underset{\_}{x},t\right)\right\rangle}_{V_{\mathrm{IRR}}}\right)\right] $$

12 $$ \frac{d{C}_{{\mathrm{Fe}}^{2+}}}{dt}=\left[\left(-{k}_1\ {C}_{{\mathrm{Fe}}^{2+}}\ {C}_{{\mathrm{H}}_2{\mathrm{O}}_2}+\gamma \right)\right]+\left[\frac{V_{\mathrm{IRR}}}{V_T}\left(\overline{\varPhi}{\left\langle \sum \limits_{\lambda }{e}_{\lambda}^a\left(\underset{\_}{x},t\right)\right\rangle}_{V_{\mathrm{IRR}}}\right)\right] $$

13 $$ \frac{d{C}_{{\mathrm{Fe}}^{3+}}}{dt}=-\frac{d\ {C}_{{\mathrm{Fe}}^{2+}}}{dt} $$

Here, ${\left\langle \sum \limits_{\lambda }{e}_{\lambda}^a\left(\underset{\_}{x},t\right)\right\rangle}_{V_{\mathrm{IRR}}}$ is the LVRPA averaged over the irradiated reactor volume (*V*_IRR_). The latter depends on the spatial photon distribution within the annular photoreactor and, consequently, on the physical properties and the geometrical characteristics of the lamp-reactor system. To compute it, a radiation model must be previously introduced. Specifically, a line source model with spherical and isotropic emission (LSSE model) was adopted (Alfano et al. 1986; Braun et al. 2004). The LSSE model allows calculating LVRPA_VIRR_ as a function of the radiation absorbing specie.

First, the following equation for the evaluation of the LVRPA for cylindrical coordinates has been solved using the numerical integration function in MATLAB:

14 $$ {e}_{\lambda}^a\left(\underset{\_}{x},t\right)={\upkappa}_{\lambda}\left(\underset{\_}{x},t\right)\frac{P_{\lambda, s}}{2\pi {L}_L}{\int}_{\uptheta_1}^{\uptheta_2}\mathit{\exp}\left[-\frac{\upkappa_{T,\lambda}\left(\underset{\_}{x},t\right)\left({r}_i-{r}_{int}\right)}{\cos \uptheta}\right] d\theta $$

where *P*_*λ*, *s*_ is the lamp spectral power emission (provided by the lamp supplier), ${\upkappa}_{\lambda}\left(\underset{\_}{x},t\right)$ is the volumetric absorption coefficient of the reacting species, ${\upkappa}_{T,\lambda}\left(\underset{\_}{x},t\right)$ is the volumetric absorption coefficient of the medium, *r is* the radius, and *L*_*L*_ is the useful length of the lamp. To compute the radiation absorbed in a generic point *I* = *I*(*r*,*z*) (located at $\underset{\_}{x}$) inside the reactor, it was necessary to estimate the limiting angles of integration (trigonometrically defined), that is:

15 $$ {\theta}_1={\tan}^{-1}\left(\frac{r_i}{L_L-{z}_i}\right)\kern0.75em \mathrm{and}\kern0.5em {\theta}_2={\tan}^{-1}\left(\frac{-{r}_i}{z_i}\right) $$

To solve Eq. (14), it was considered that ferric ions present in solution as ferric ion complex (Fe(OH)^2+^) are the dominant ferric species at pH 2.8 and the principal absorbing specie; here it was also assumed that radiation absorption of hydrogen peroxide and ferrous ion is negligible for wavelengths greater than 300 nm. Under these hypotheses, ${\upkappa}_{T,\lambda}\left(\underset{\_}{x},t\right)$ can be calculated as follows:

16 $$ {\upkappa}_{T,\lambda}\left(\underset{\_}{x},t\right)=\sum \limits_i{\alpha}_{i,\lambda }{C}_i\overset{\sim }{=}{\alpha}_{\mathrm{Fe}{\left(\mathrm{OH}\right)}^{2+},\lambda }\ {C}_{\mathrm{Fe}{\left(\mathrm{OH}\right)}^{2+}} $$

where ${\alpha}_{\mathrm{Fe}{\left(\mathrm{OH}\right)}^{2+},\lambda }$ is the molar absorptivity of ferric ion complex (Fe(OH)^2+^) and ${C}_{\mathrm{Fe}{\left(\mathrm{OH}\right)}^{2+}}$ is the concentration of the latter that can be considered equal to the concentration of Fe^3+^.

Finally, after evaluating LVRPA at each point inside the irradiated volume, it is possible to compute the averaged value of the LVRPA over the irradiated reactor volume and polychromatic radiation, solving the following equation:

17 $$ {\left\langle \sum \limits_{\lambda }{e}_{\lambda}^a\left(\underset{\_}{x},t\right)\right\rangle}_{V_{IRR}}=\frac{2\pi }{V_{\mathrm{IRR}}}{\int}_0^L{\int}_{r_{int}}^{r_{ext}}{e}_{\lambda}^a\left(\underset{\_}{x},t\right)\ r\  dr\  dz $$

where *r*_*int*_ and *r*_*ext*_ are the internal and external radius of the annular photoreactor. Also in this case, the numerical integration function in MATLAB was used to solve Eq. (17).

In this way, it was possible to estimate the value of LVPRA averaged over the irradiated reactor volume for a specific set of values of ${C}_{{\mathrm{Fe}}^{3+}}$ (Table 5).

**Table 5:** Values of LVRPA averaged over the irradiated reactor volume, calculated for a specific set of iron concentration

${C}_{{\mathrm{Fe}}^{3+}}$ (mg L^−1^)	${\left\langle \sum \limits_{\lambda }{e}_{\lambda}^a\left(\underset{\_}{x},t\right)\right\rangle}_{V_{\mathrm{IRR}}}$(Einstein cm^−3^ s^−1^)
0.0	0
2.5	3.5 × 10^−10^
5.0	7.1 × 10^−10^
7.5	9.5 × 10^−10^
10.0	1.18 × 10^−9^

### Model fitting and parameter estimation

A nonlinear multivariate and multiparameter optimization procedure was implemented in MATLAB in order to minimize the sum of the squared differences between the experimental and model values of PCT and H_2_O_2_ normalized concentrations.

**Fig. 2:**
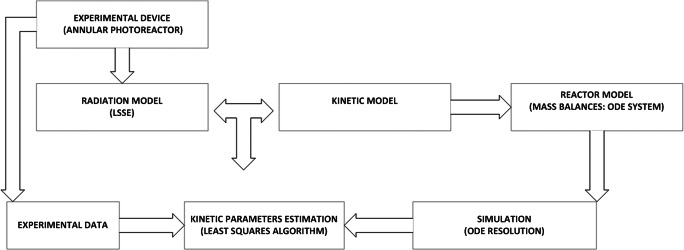
Methodological framework followed to estimate the parameters of the proposed kinetic model

The first step is to solve numerically the ODE system given by Eqs. (8)–(9). For this purpose, an ordinary differential equation (ODE) solver in MATLAB was used. Especially, ode15s solver stiff differential equations which is a variable-step, variable-order (VSVO) solver based on the numerical differentiation formulas (NDFs), was selected. Then, the values of the kinetic constants (*k*_1_, *k*_3_, *k*_4_, and *k*_5_) were estimated minimizing the sum of the squared differences between the model values (calculated by solving the ODEs system) and the experimental values of PCT and H_2_O_2_. For this purpose, the Levenberg-Marquardt least-squares algorithm available in the optimization toolbox of MATLAB was used. The whole set of experimental data (E1-E18*,* Table 3) was used for the parameter estimation. Additionally, the root mean square errors (RMSE) were calculated to test model reliability (see Fig. 2).

## Results and discussion

### Experimental results

Paracetamol was not detected by the HPLC analyses under all investigated conditions after a maximum time of 15 min (E1, Table 3) and a minimum time of 2.5 min (E18, Table 3) (results not shown). Especially, for the highest value of ${C}_{{\mathrm{H}}_2{\mathrm{O}}_2}^{t0}$, and considering dark conditions and increasing ${C}_{{\mathrm{Fe}}^{2+}}^{to}$ from 5, to 7.5 and 10 mg L^−1^ (experiments E3, E6 and E9, Table 3), PCT total removal was obtained in 15, 10, and 5 min, respectively. In addition, for this dark conditions and considering the minimum concentration of oxidizing agent (${C}_{{\mathrm{H}}_2{\mathrm{O}}_2}^{t0}=94.5\ \mathrm{ppm}$), the reaction time necessary to achieve complete PCT destruction is reduced by 66% by doubling the initial catalyst concentration from 5 to 10 ppm (E1 and E7 tests respectively, Table 3). On the other hand, for the highest value of ${C}_{{\mathrm{H}}_2{\mathrm{O}}_2}^{t0}$ and irradiating system (experiments E12, 15 and E18, Table 3), the PCT total conversion was obtained in only 10, 7.5, and 2.5 min, respectively. Therefore, considering irradiated conditions, by doubling the initial concentration of the catalyst, it is possible to reduce by up to 75% the time necessary for the complete destruction of the contaminant. Therefore, it was possible to confirm the beneficial effect of using higher doses of catalyst to reduce the reaction times necessary to achieve complete removal of the contaminant. This effect is of greater relevance for lower concentrations of oxidizing agent.

**Fig. 3:**
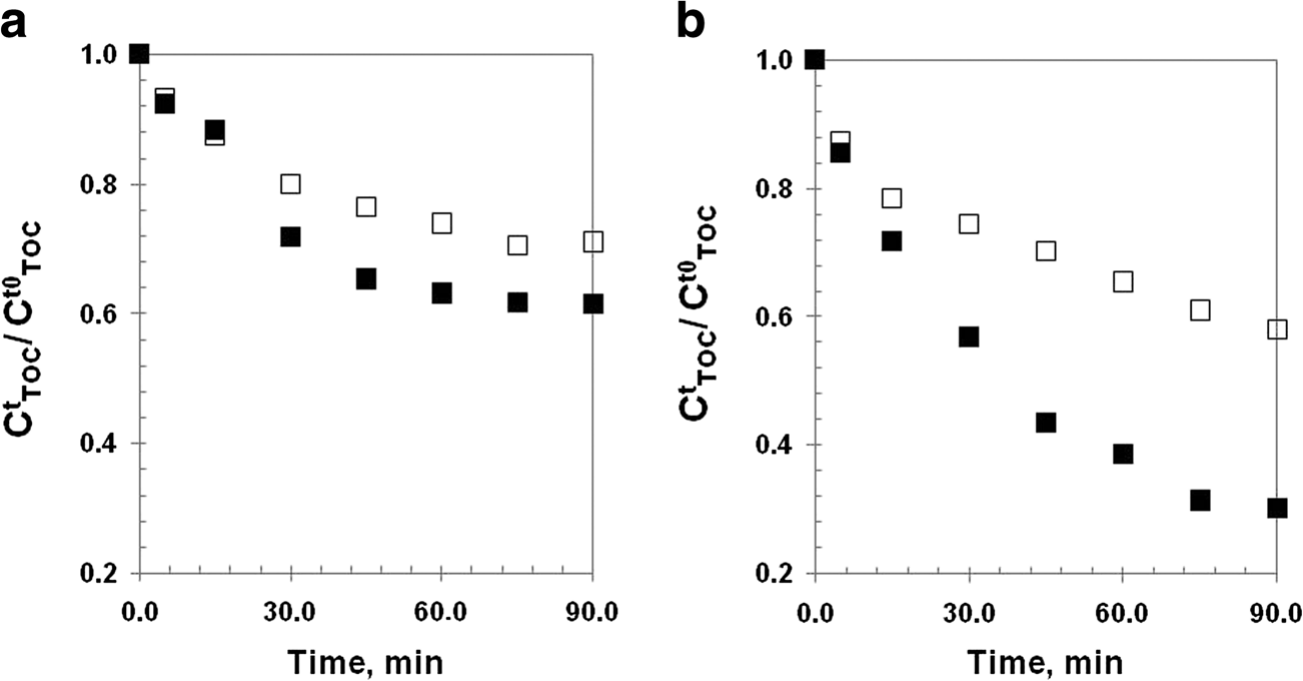
Normalized experimental concentrations of TOC, obtained under dark (□) and irradiated(■) conditions and considering the lowest and highest initial concentrations of Fe^2+^ and H_2_O_2_$\Big({C}_{{\mathrm{Fe}}^{2+}}^{to}$ and ${C}_{{\mathrm{H}}_2{\mathrm{O}}_2}^{t0}$): experiments E1 and E10 (**a**) and experiments E9 and E18 (**b**), respectively

Although TOC was not introduced as a kinetic model component, it represents an important measurement to analyze the process performance. Hence, the performance of each experiment was also evaluated in terms of reached mineralization levels at a specific time (TOC conversion,${\mathrm{X}}_{\mathrm{TOC}}^{\mathrm{t},\min }$). In Fig. 3, the TOC experimental evolution obtained using the minimum (Fig. 3a) and maximum (Fig. 3b) concentrations of oxidizing agent and catalyst is shown.

In Fig. 3a, it can be observed that TOC conversion reaches asymptotic values in both experiments, specifically, ${X}_{\mathrm{TOC}}^{75\ \mathit{\min}}=29.3\%$ and ${X}_{\mathrm{TOC}}^{60\ \mathit{\min}}=33\%$ for E1 and E10, respectively. However, these maximum conversion levels are associated with reaction times in which the oxidant agent is no more detected (75 and 60 min for E1 and E10, respectively). Although the TOC conversions obtained in both cases are similar, the use of radiation allowed reducing the reaction time by 20%. Therefore, even in the case of achieving the total removal of the contaminant (15 min of reaction) with the minimum concentrations of reagents, the level of mineralization reached is not significant. This is in accordance with the theoretical levels of oxidant agent doses required to achieve complete mineralization of the system (see Eq. (1)).

Furthermore, in Fig. 3b, it can be appreciated an asymptotic value of ${X}_{\mathrm{TOC}}^{75\ \mathit{\min}}=68.5\%$ (depletion of H_2_0_2_) in the case of the assay E18. This value was the highest level of mineralization that was reached for the whole set of performed experiments. Nevertheless, under the same maximum concentrations of the reagents, the dark reaction (E9), for which H_2_O_2_ was detected until the end of the assay (120 min), only reached ${X}_{\mathrm{TOC}}^{120\ \mathit{\min}}=45.6\%$.

Finally, in order to conclude the study regarding the mineralization performance of the system, the specific consumption of the oxidizing agent, ${\varUpsilon}_{{\mathrm{H}}_2{\mathrm{O}}_2/\mathrm{TOC}}$, was evaluated as follows:

18 $$ {\varUpsilon}_{H_2{O}_2/ TOC}=\frac{C_{H_2{O}_2}^{t0}-{C}_{H_2{O}_2}^{tf}}{C_{TOC}^{t0}-{C}_{TOC}^{tf}} $$

where *t*_*f*_ is the time at which the oxidizing agent consumption occurred or the final reaction time.

**Fig. 4:**
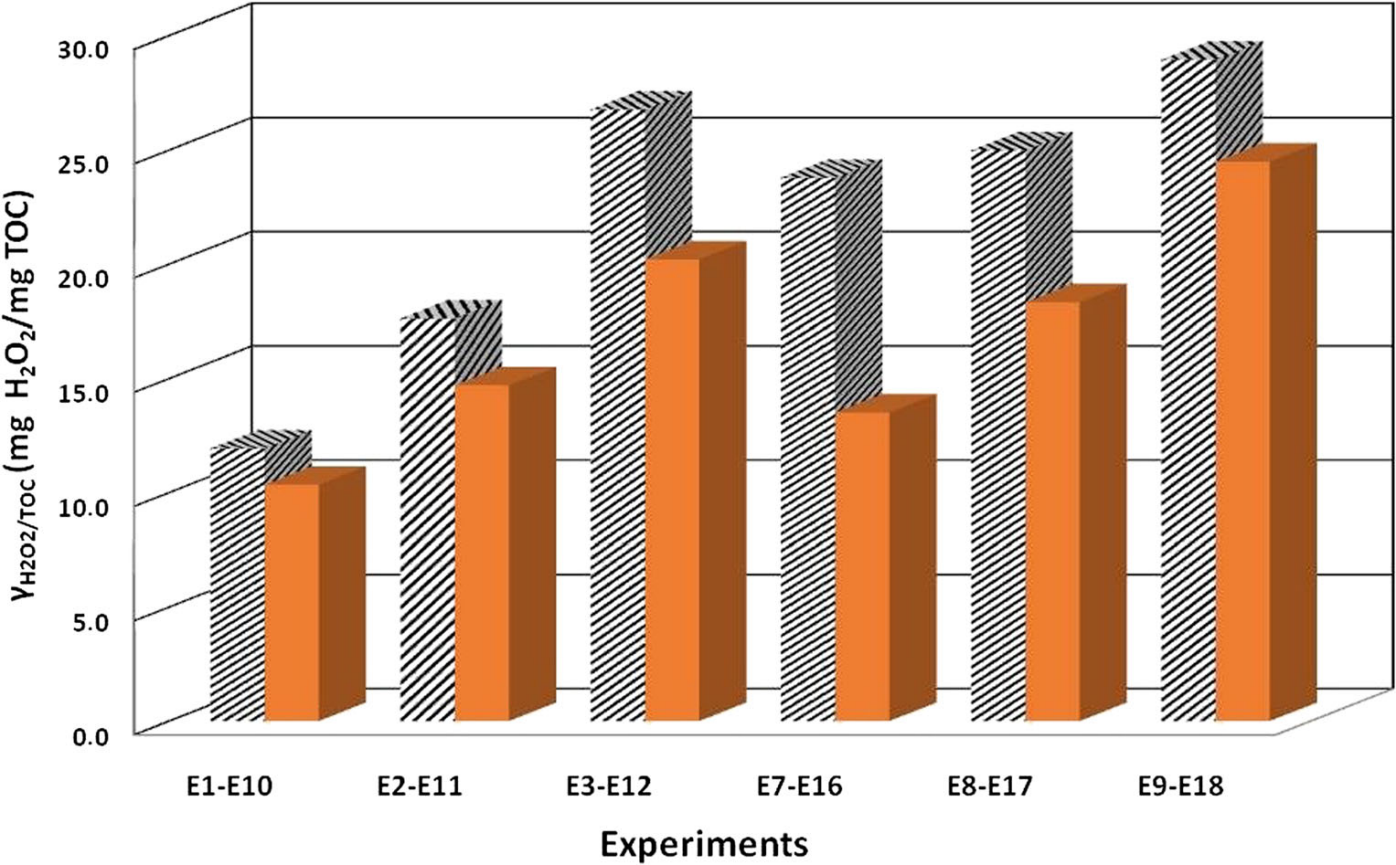
Specific consumption of the oxidizing agent as a function of the experiments performed with ${C}_{{\mathrm{Fe}}^{2+}}^{to}$ = 5 and 10 mg L^−1^ and for the three different investigated values of ${C}_{{\mathrm{H}}_2{\mathrm{O}}_2}^{t0}$ (94.5, 189, 378 mg L^−1^) under nonirradiated (striped bar chart) and irradiated conditions (solid bar chart)

In Fig. 4, a multiple bar chart shows the radiation effect on ${\varUpsilon}_{H_2{O}_2/ TOC}$ obtained for several initial concentrations of the oxidant (94.5, 189 and 378 mg L^−1^) and of the catalyst (5 and 10 mg L^−1^).

First of all, it must be noticed that for all the investigated conditions, the ${\varUpsilon}_{H_2{O}_2/ TOC}$ results are always higher for non-irradiated conditions than for irradiated ones. This result highlights a less efficient consumption of H_2_O_2_ under non-irradiated conditions. Moreover, it is possible to observe that the increase in the initial concentration of the catalyst led to an increase of the gap between the H_2_O_2_ specific consumption obtained under non-irradiated and irradiated conditions.

It is worth noticed that, although experiment E10 (${C}_{{\mathrm{Fe}}^{2+}}^{to}$ = 5 mg L^−1^ and ${C}_{{\mathrm{H}}_2{\mathrm{O}}_2}^{t0}$ = 94.5 mg L^−1^), carried out under irradiated conditions, showed to be the most efficient one, only allowed to reach a final TOC conversion of 39%. However, experiment E12 (${C}_{{\mathrm{Fe}}^{2+}}^{to}$ = 5 mg L^−1^ and ${C}_{{\mathrm{H}}_2{\mathrm{O}}_2}^{t0}$ = 378 mg L^−1^), also performed under irradiated conditions, despite being less efficient than E10, allowed to obtain a final TOC conversion of 74%. This result presents great environmental and economic significance since, according to the level of mineralization required by the system, it would allow knowing the dose of oxidizing agent and necessary reaction time. Hence, this result shows that, if from one hand, the increase of the initial concentration of the oxidant led to a less efficient consumption of this last, from the other hand it allows reaching higher levels of mineralization.

### Model fitting

The experimental data were used to perform the fitting of the proposed kinetic model. The values of the kinetic parameters accounting for the Fenton and Fenton-like reactions (*k*_1_ and *k*_3_ respectively) and for hydroxyl radical attack to hydrogen peroxide and paracetamol (*k*_4_ and *k*_5_ respectively) were estimated and are shown in Table 6.

**Table 6:** Estimated values of the kinetic parameters

*k*_1_	*k*_3_	*k*_4_	*k*_5_
M^−1^ s^−1^	M^−1^ s^−1^	M^−1^ s^−1^	M^−1^ s^−1^
147.29	3.16	7.00 × 10^7^	3.58 × 10^9^

It is worth noting that the estimated values of the kinetic parameters *k*_4_ and *k*_5_ are within the range of values found in the specific literature (Simunovic et al. 2011, De Laurentiis et al. 2014). Conversely, the values of the kinetic parameters *k*_1_ and *k*_3_ result to be slightly higher than those found in the literature (63–76 M^−1^ s^−1^ and 0.01–0.02 M^−1^ s^−1^, respectively) (Simunovic et al. 2011).

**Fig. 5:**
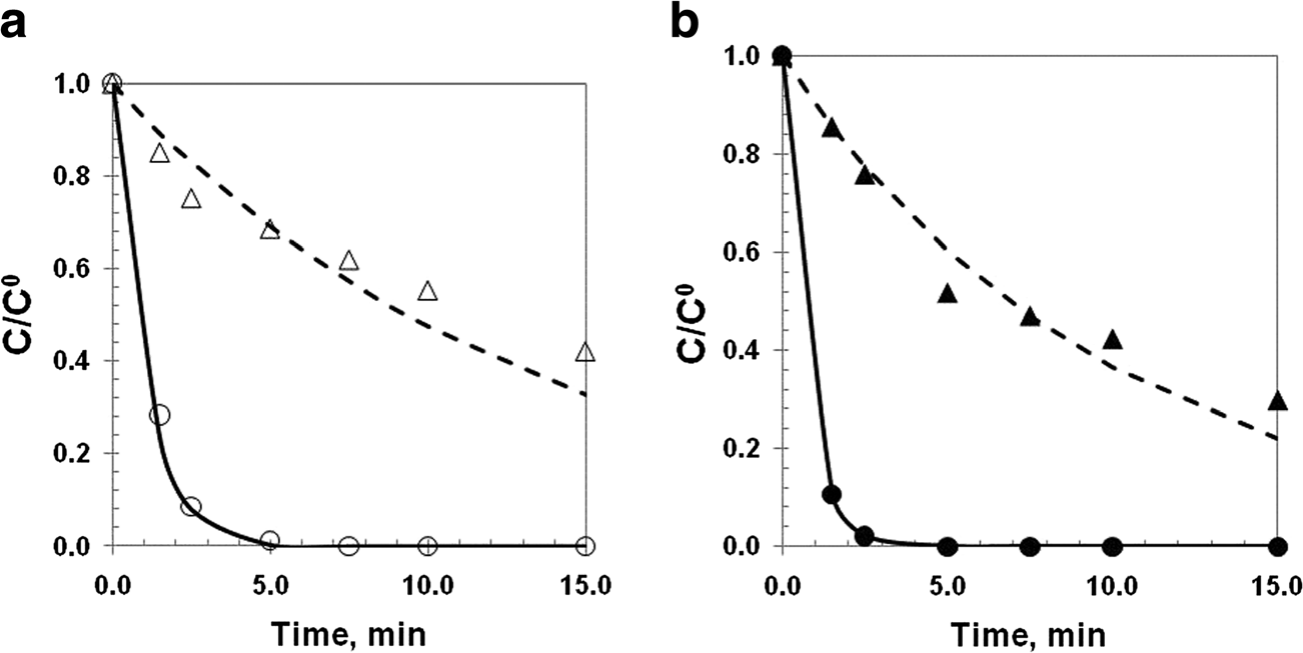
Experimental concentrations of paracetamol (○,●) and hydrogen peroxide (Δ,▲) and predicted (▬) and hydrogen peroxide (- -). Dark (○,Δ) and irradiated (●,▲) conditions. a Experiment E6 (${C}_{{\mathrm{Fe}}^{2+}}^{to}$= 7.5 mg L^−1^ and ${C}_{{\mathrm{H}}_2{\mathrm{O}}_2}^{t0}$ = 378 mg L−^1^), and b experiment E18 ${C}_{{\mathrm{Fe}}^{2+}}^{to}$= 10 mg L^−1^ and ${C}_{{\mathrm{H}}_2{\mathrm{O}}_2}^{t0}$ = 378 mg L^−1^)

In Fig. 5, the comparison between experimental and predicted concentrations of H_2_O_2_ and PCT obtained for dark (Fig. 5a) and irradiated conditions (Fig. 5b), is shown.

Firstly, using the same initial concentration of H_2_O_2_ under dark conditions and moving from ${C}_{{\mathrm{Fe}}^{2+}}^{to}$ = 7.5 mg L^−1^ (Fig. 5a) to ${C}_{{\mathrm{Fe}}^{2+}}^{to}$ = 10 mg L^−1^ (data not shown), it was observed that the time at which PCT is no more detected by HPLC remained the same, approximately 5 min in both cases. However, it was observed that the irradiated condition (Fig. 5b) led to the complete PCT removal in only 2.5 min. Hence, under these experimental conditions, the photo-Fenton process allowed to reach a 50% decrease in the total PCT removal time.

In order to test the model reliability, the root mean square error (RMSE) was calculated by the expression:

$$ {\displaystyle \begin{array}{l}\begin{array}{l} RMS{E}_i=\sqrt{\sum \limits_k{\left({y}_{ik}-{y}_{ik}^{\ast}\right)}^2/{n}_i},\mathrm{root}\ \mathrm{mean}\ \mathrm{square}\ \mathrm{error}\ \mathrm{of}\ \mathrm{the}\ {i}^{\mathrm{th}}\ \mathrm{variable}\\ {}{y}_{ik},\mathrm{value}\ \mathrm{of}\ \mathrm{the}\ {k}^{\mathrm{th}}\ \mathrm{measurement}\ \mathrm{of}\ \mathrm{the}\ {i}^{\mathrm{th}}\ \mathrm{variable}\\ {}{y}_{ik}^{\ast },\mathrm{value}\ \mathrm{of}\ \mathrm{the}\ {k}^{\mathrm{th}}\ \mathrm{estimation}\ \mathrm{of}\ \mathrm{the}\ {i}^{\mathrm{th}}\ \mathrm{variable}\ \left(\mathrm{model}\ \mathrm{prediction}\right)\\ {}i=1,2,\dots I,{i}^{\mathrm{th}}\ \mathrm{element}\ \mathrm{of}\ \mathrm{the}\ \mathrm{s}\mathrm{et}\ \mathrm{of}\ \mathrm{measured}\ \mathrm{variable}\mathrm{s}\\ {}k=1,2,\dots {n}_i,{k}^{\mathrm{th}}\ e\mathrm{lement}\ \mathrm{of}\ \mathrm{the}\ \mathrm{s}\mathrm{et}\ \mathrm{of}\ \mathrm{measurements}\ \mathrm{of}\ \mathrm{variable}\kern0.50em i,\end{array}\\ {}\mathrm{The}\ \mathrm{only}\ \mathrm{measurements}\ \mathrm{considered}\ \mathrm{are}\ \mathrm{the}\ \mathrm{normalized}\ \mathrm{concentration}\ \mathrm{of}\ \mathrm{PCT}\ \mathrm{and}\ {\mathrm{H}}_2{\mathrm{O}}_2\left(I=2\right).\end{array}} $$

In Table 7, the values of RMSE, calculated considering: (i) the whole set of experiments (E1 to E18), (ii) the experiments performed only under dark conditions (E1 to E9), and (iii) the experiments performed only under irradiated conditions (E10 to E18), are shown.

**Table 7:** Root mean square errors (RMSE)

	Dark and irradiated conditions	Dark conditions	Irradiated conditions
PCT	6.80%	7.64%	5.84%
H_2_O_2_	9.67%	9.75%	9.59%

The proposed model showed a relatively good agreement with the experimental data. The lowest values of RMSE (for normalized PCT and H_2_O_2_ concentration) were obtained in case of considering only the experiments carried out under irradiated conditions. However, considering the complete set of performed tests, the obtained errors are consistent (*RMSE*_PCT_ = 6.80% and *RMSE*_H2O2_ = 9.67%) and, therefore, the kinetic model is able to satisfactorily reproduce the system behavior.

## Conclusions

The Fenton and photo-Fenton degradation of paracetamol and the consumption of hydrogen peroxide have been investigated in a well-stirred annular reactor placed inside the loop of a batch recycling system. Total removal of paracetamol was achieved for all the analyzed operating conditions, with a maximum removal time of 15 min. Moreover, for each concentration of iron and oxidizing agent, no significant differences were observed in the removal times required for both dark and irradiated tests. However, it should be mentioned that the reaction times were always lower for the irradiated operating conditions, with a minimum required time of 2.5 min.

In addition, the use of radiation allowed a significant enhancement of the process performance leading to a more efficient consumption of the oxidizing agent. For all the evaluated operating conditions, the values of the “specific consumption of the oxidant agent” (${\varUpsilon}_{H_2{O}_2/ TOC}$) obtained for Fenton process were always higher than the corresponding values observed for photo-Fenton system.

Furthermore, the performance of each experiment was also evaluated in terms of reached mineralization levels at a specific reaction time. The highest level of mineralization achieved considering the whole set of experiments was ${X}_{\mathrm{TOC}}^{75\ \mathit{\min}}=68.5\%$, conversion value obtained at the time when the oxidizing agent had been completely consumed.

A kinetic model for predicting Fenton and photo-Fenton degradation of paracetamol and hydrogen peroxide consumption has been proposed. Results have shown that the proposed kinetic model is able to capture the complex and nonlinear nature of such processes and incorporate the effect of the local volumetric rate of photon absorption on the reactor behavior.

Kinetic parameters, accounting for the Fenton and photo-Fenton reaction and the *HO*^∙^attack to PCT and H_2_O_2_ were estimated. Considering the values of the root mean square error (RMSE) obtained for the complete set of experimental runs (*RMSE*_PCT_ = 6.80% and *RMSE*_H2O2_ = 9.67%), it was possible to conclude that the proposed kinetic model is able to satisfactorily reproduce the system behavior.

This kinetic model is the starting point for the development of a more complex model that also takes into account the generation of intermediate compounds and the evolution of the total organic carbon, and their main effects on the reaction rate of the photo-Fenton system.
