# Correction to: A kinetic study for the Fenton and photo-Fenton paracetamol degradation in an annular photoreactor

**DOI:** 10.1007/s11356-021-15050-7

**Published:** 2021-07-01

**Authors:** Francesca Audino, Leandro Oscar Conte, Agustina Violeta Schenone, Montserrat Pérez-Moya, Moisès Graells, Orlando Mario Alfano

**Affiliations:** 1 Chemical Engineering Department, Universitat Politècnica de Catalunya, Escola d’Enginyeria de Barcelona Est (EEBE), Av. Eduard Maristany, 16, 08019 Barcelona, Spain; 2 Consejo Nacional de Investigaciones Científicas y Técnicas (CONICET) and Universidad Nacional del Litoral (UNL), Instituto de Desarrollo Tecnológico para la Industria Química (INTEC), 3000 Santa Fe, Argentina

**Correction to: Environmental Science and Pollution Research (2019) 26:4312–4323**

10.1007/s11356-018-3098-4

The article A kinetic study for the Fenton and photo-Fenton paracetamol degradation in an annular photoreactor, written by Francesca Audino, Leandro Oscar Conte, Agustina Violeta Schenone, Montserrat Pérez-Moya, Moisès Graells and Orlando Mario Alfano, was originally published electronically on the publisher’s internet portal on 18 September 2018 without open access. With the author(s)’ decision to opt for Open Choice the copyright of the article changed on 14 June 2021 to © The Author(s) 2021 and the article is forthwith distributed under a Creative Commons Attribution 4.0 International License, which permits use, sharing, adaptation, distribution and reproduction in any medium or format, as long as you give appropriate credit to the original author(s) and the source, provide a link to the Creative Commons license, and indicate if changes were made. The images or other third party material in this article are included in the article’s Creative Commons license, unless indicated otherwise in a credit line to the material. If material is not included in the article’s Creative Commons license and your intended use is not permitted by statutory regulation or exceeds the permitted use, you will need to obtain permission directly from the copyright holder. To view a copy of this license, visit http://creativecommons.org/licenses/by/4.0.

The Original article has been corrected.

